# Parental Burden and its Correlates in Families of Children with Autism Spectrum Disorder: A Multicentre Study with Two Comparison Groups

**DOI:** 10.2174/1745017901814010143

**Published:** 2018-07-31

**Authors:** Angelo Picardi, Antonella Gigantesco, Emanuele Tarolla, Vera Stoppioni, Renato Cerbo, Maurizio Cremonte, Guido Alessandri, Ilaria Lega, Franco Nardocci

**Affiliations:** 1 Centre for Behavioural Sciences and Mental Health, Italian National Institute of Health, Rome. Italy; 2 Department of Mental Health, Local Health Unit, Trento, Italy; 3 Department of Pediatrics and Child and Adolescent Neuropsychiatry, Marche Nord Hospital, Fano, Italy; 4 Child Neuropsychiatry Unit and Centre for Neurodevelopmental Disorders, Pescara, Italy; 5 Neurological and Psychiatric Child Unit, Pediatric Department, Alessandria Hospital, Alessandria, Italy; 6 Department of Psychology, Sapienza University of Rome, Italy; 7 Women’s Health Unit, National Centre of Epidemiology Surveillance and Health Promotion, Italian National Institute of Health, Rome. Italy; 8 Italian Society for Child and Adolescent Neuropsychiatry, Italy; 9 Italian Autism Foundation, Rome, Italy

**Keywords:** Autism Spectrum Disorders, Parental burden, Psychological distress, Coping, Resilience, Social support

## Abstract

**Background:**

The effects of having a child with Autism Spectrum Disorder (ASD) on parents are multifaceted and pervasive. While ample evidence has been provided that these families are under severe stress, there are still several knowledge gaps and unresolved questions.

**Objective:**

This study aimed at quantifying the subjective and objective burden of ASD in mothers and fathers, and at improving the understanding of the interplay between parental burden, child’s characteristics, and parents’ coping resources and strategies.

**Methods:**

The parents of 359 children/adolescents with ASD were compared to parents of age-matched patients with Down syndrome (N=145) and Type 1 diabetes mellitus (N=155). Child’s clinical characteristics and parents’ caregiving burden, psychological distress, coping resources and strategies were assessed.

**Results:**

The parents of children with ASD reported higher objective and subjective burden, more frequent psychological distress, lower social support. Mothers reported greater subjective burden than fathers. Structural equation modeling showed that the most consistent positive and negative predictors of objective and subjective burden were ASD symptom severity and social support, respectively. Other positive predictors were engagement, distraction and disengagement coping, intellectual disability, and adaptive functioning. Other negative predictors were spiritual wellbeing and hardiness. Some effects were indirect through social support and coping strategies.

**Conclusion:**

This study confirmed that parents of children with ASD carry a huge caregiving burden, and added to our understanding of the factors associated with burden. The findings may help inform the design of effective interventions aimed at reducing burden among the parents of children with ASD.

## 1 INTRODUCTION

Autism Spectrum Disorders (ASD) include a variety of childhood-onset and lifelong neurodevelopmental disorders with an enduring impact on multiple domains of functioning, characterized by persistent deficits in social communication and social interaction and restricted and repetitive behaviour, interest and activities [1]. A recent review of epidemiological surveys of autistic disorder and pervasive developmental disorders estimated a global median prevalence rate of ASD of 62/10,000 in the general population worldwide [2]. Even referring to the most conservative prevalence estimates, ASD is among the world's 20 most disabling childhood conditions, with Autistic Disorder accounting for more than 58 DALYs per 100,000 population and other ASD for 53 DALYs per 100,000 [3].

The effects of having a child with ASD on parents and families are, like the disorder itself, multifaceted and pervasive. Meeting the high care demands of affected children requires much time, effort and patience. Caring for children with ASD is challenging due to the severity and chronicity of ASD, their extensive developmental and physical comorbidities, and the difficulties of health services in making widely available the integrated and intensive interventions needed by persons with ASD [4]. The huge impact of having a child with ASD is apparent in both the severity and breadth of parent domains that seem to be influenced [5].

The personal suffering of a caregiver as a consequence of the illness of a family member is termed ‘burden’ [6]. Commonly, the literature distinguishes between objective burden, which refers to practical problems (*e.g*., disturbed family relationships; constraints in social, leisure and work activities; financial difficulties), and subjective burden, which refers to caregivers’ psychological reactions (*e.g*., loss of hope, dreams, and expectations; depression; anxiety; embarrassment in social situations) [7].

One of the most widely examined areas of subjective burden among families of children with ASD is parenting stress, which can be described as the experience of distress that derives from the demands that parenting implies, and is usually measured with specific parent self-report questionnaires. A recent meta-analysis [8] found that caring for a child with ASD is associated with greater parenting stress, both when families of a child with ASD are compared to families of a child with typical development, and when they are compared to families of a child diagnosed with another disability, such as Down syndrome, cerebral palsy, and intellectual disability. The overall effect size calculated was large for both the analyses.

In addition to experiencing substantial levels of stress, the parents of children with ASD are at increased risk for mental health concerns, as they displayed high levels of depressive symptoms [9-11] and psychological distress [12]. Moreover, compared to parents of children with intellectual disability, developmental delay, behavioural disorders, Down syndrome, or typically developing children, they showed increased levels of depression [13-17], anxiety [14-16], psychological distress [18], and general psychopathology [19].

Regarding objective burden, these families face a multitude of practical problems and demands, including continuous time pressures, greater necessity for vigilant parenting, the need to provide support and accommodations for their child’s education, greater investment in healthcare, constant self- and child-advocacy, less opportunities to work, and a higher rate of divorce as compared with families with typically developing children [5]. Compared with mothers of children without disabilities, the mothers of adolescents and adults with ASD were found to experience more fatigue and to spend more time providing childcare and doing household chores, and less time in leisure activities [20]. Moreover, parents of children with ASD endure significant financial burden, in the form of high out-of-pocket healthcare expenses, underemployment, or employment loss [21-25].

In recent years, the literature on burden and stress in families of children with ASD has expanded greatly, and ample evidence has been provided that these families are under severe stress. The most important research question now revolves around the identification of the factors that may contribute to, or alleviate stress and burden in these families [8]. Improved knowledge of these factors would facilitate the development of more targeted interventions to provide relief to families.

Previous research focusing on the relationship between child characteristics and parental burden did not provide consistent findings. Several child variables, including age [26-29], severity of ASD symptoms [9, 12, 30-37], adaptive behaviour [31, 37-39], and intellectual disability [40, 41] have been associated with parental stress or burden in caregivers of children with ASD. However, some studies yielded negative results regarding age [42-44], ASD symptom severity [43, 45-48], cognitive impairment [9, 31, 49], and adaptive skills [14, 42, 50].

Besides child characteristics, parents’ characteristics may also affect burden. The most relevant are parents’ coping resources upon which they may draw upon when confronted with stress, such as social support and some personal characteristics, and parent’s coping strategies, *i.e*., the behavioural and cognitive attempts used to manage stressful situational demands [51].

Despite the theoretical importance of the topic, research on coping strategies among parents of children with ASD has been relatively scarce. The few studies performed suggested that parental stress is positively correlated with strategies based on distraction and disengagement [18, 28, 47, 52, 53], and negatively correlated with strategies such as positive reframing and acceptance [47, 48].

Concerning social support, with only one exception [18], all studies reported a link between this resource and lower levels of burden in parents of children with ASD [12, 31, 35, 54-58]. However, most studies focused on the association between social support and burden, rather than on differences in social support between families of children with ASD compared to other families.

Personal resources, such as spirituality and resilience, may also be important. Resilience is indeed increasingly recognized as a topic of interest in family research as a means to emphasize healthy family functioning in the face of chronic stress. However, personal characteristics associated with resilience, such as hardiness, sense of coherence, and internal locus of control have only rarely been included in studies of burden in families of children with ASD [16, 56, 59, 60].

The literature overview suggests there are still some gaps and unresolved questions. First, previous findings have been inconsistent concerning the relationship between parental burden and child’s characteristics, such as age, sex, autism symptom severity, intellectual disability, and adaptive functioning. Second, few studies have investigated differences in social support and coping strategies between families of children with ASD and other families. Third, research on coping resources besides social support, such as hardiness and spiritual wellbeing, is scarce. Fourth, most studies focused either on the subjective or objective burden, rather than both aspects simultaneously. Fifth, most studies examined the association between parental burden and one or a few factors, rather than a comprehensive set of child and parent factors. Sixth, research is unclear about the differences between mothers and fathers; most studies were performed on mothers only, and even in those that included both parents, mothers were often overrepresented, and the results were frequently reported without differentiating between mothers and fathers [8]. Finally, a crucial issue is how child and parent variables may interact with each other to increase or decrease parental burden. These interactions may involve not only direct but also indirect effects. For instance, a greater impairment in child’s functioning or more severe autistic symptoms may make it more difficult for parents to maintain social interactions with family and friends, which may lead to increased burden through reduced social support. Moreover, the parents with greater spiritual well-being may perceive their social support to be greater and may also actually elicit more support from others, which, in turn, may lead to a lower burden. Greater spiritual wellbeing and acceptance may also be associated with lower use of problem-focused coping strategies aimed at changing the situation, which may not produce the desired results and even be counterproductive in the face of chronic and difficult to manage problems such as autistic symptoms in a child [48]. Similarly to spiritual wellbeing, greater parental hardiness may also be associated with greater social support. On the other hand, the strong sense of control and mastery over life that is typical of individuals high in hardiness may also be associated with more strenuous coping efforts, which may lead to a greater burden.

This study aimed at addressing the literature gaps mentioned above, and at improving the understanding of the interplay between parental burden, child characteristics, and parents’ coping resources and strategies. The mothers and fathers of children and adolescents with ASD were compared to the mothers and fathers of age-matched children and adolescents with either a condition that brings with it the challenge of intellectual disability, such as Down Syndrome (DS), or a potentially life-threatening condition requiring continuous medical treatment, such as Type 1 Diabetes Mellitus (T1DM). The rationale for this choice was twofold. First, it allowed us to disentangle the influence of social and communicative disability on burden from the influence of intellectual disability and the non-specific effects of any serious chronic illness, such as limitations on family opportunities, financial strains and increased caretaking demands. Second, the comparison of families facing different conditions and challenges allowed us to investigate group differences and similarities in how child and parent characteristics interact in increasing or buffering burden.

To provide some context for the study, in Italy healthcare is provided to the entire population by the National Health Service (NHS), which has a similar structure to that of the British NHS. All citizens have access to unlimited health care coverage through ‘Local Health Units’, each of which is responsible for a geographically defined catchment area. Access to health services is generally free of charge, although some fees are charged for specific medical examinations; medicines for major diseases are generally free of charge or available at small cost.

## 2 METHODS

### 2.1 Study Design and Preliminary Phases

This study was a multicentre cross-sectional study coordinated by the Italian National Institute of Health (INIH), in which three main collaborating centres were involved: the Child Neuropsychiatric Unit of the Arrigo Hospital in Alessandria (North Italy), the Centre of Autism and Developmental Disorders in Ravenna (Central Italy) and the Autism Regional Reference Centre in L’Aquila (Southern Italy). The study was formally approved by the Ethics Committee of the INIH, which provides an evaluation of research proposals according to current EU and national legislation, Helsinki Declaration and Council for International Organizations of Medical Sciences, International Ethical Guidelines for Biomedical Research.

Each collaborating centre identified from 3 to 6 health districts in the respective geographical area (Northern, Central, or Southern Italy). In Italy, the National Health Service provides health care to the entire population, and all citizens have access to unlimited health care coverage through health districts, each of which manages a geographically defined catchment area, which may encompass one large town or two small towns. Overall, 12 health districts were identified: 3 in Northern Italy, 6 in Central Italy, and 3 in Southern Italy. In each of these districts, a number of Children and Adolescent Neuropsychiatric Units and Paediatric Units were selected as recruitment sites and were involved in the study.

### 2.2 Participants

Drawing from the population of patients with the conditions of interest attending each unit, the participating units recruited three groups of consecutive child and adolescent patients and their parents. The first group included families of children/adolescents who met DSM-IV-TR [61] criteria for ASD (Autism, or Asperger's Syndrome, or Pervasive Developmental Disorder Not Otherwise Specified). To be included in the study, patients had to meet the following criteria: duration of illness of at least 12 months; current age between 5 and 17 years; diagnosis confirmed by expert clinical evaluation and Autism Diagnostic Observation Schedule (ADOS); not suffering from coexisting DS, T1DM, sensory disorders (either visual or auditory), or motor disorders.

The second group comprised families with children/adolescents who suffered from DS. In order to be included in the study, patients had to meet the following criteria: duration of illness of at least 12 months; current age between 5 and 17 years; not suffering from coexisting ASD, T1DM, other severe psychiatric disorders, sensory disorders (either visual or auditory), motor disorders or significant autistic traits as indicated by a Child Autism Rating Scale (CARS) score lower than 30.

The third group included families with children/adolescents diagnosed with T1DM. To be included in the study, patients had to meet the following criteria: duration of illness of at least 12 months; current age between 5 and 17 years; not suffering from coexisting ASD, DS, intellectual disability, other severe psychiatric disorders, sensory disorders (either visual or auditory), or motor disorders.

The inclusion criteria for parents in all the study groups were the following: Italian nationality; absence of intellectual disability, dementia, psychotic disorders, bipolar disorder, substance use disorders, or severe medical illness (*e.g*., cardiovascular or pulmonary diseases); not having another child/adolescent with mental illness or chronic medical illness in the same household.

The recruitment period lasted from February 2010 to February 2011. In each geographical area, the sampling was stratified by health district and age group (5-8, 9-12 and 13-17 years), with a ratio of cases (ASDs) to controls (T1DM and DS) of 2:1. A total of 659 families were recruited, of which 359 belonged to the ASD group (351 mothers, 288 fathers), 155 to the T1DM group (153 mothers, 133 fathers), and 145 to the DS group (140 mothers, 115 fathers). Recruitment was balanced across sites, except for a lower number of families recruited (especially in the T1DM group) in Northern Italy, where a sad circumstance, the untimely death of the local study coordinator, caused organisational difficulties and delays.

### 2.3 Procedure

A research assistant (either a child/adolescent psychiatrist or psychologist with experience in clinical research and trained in the use of the study instruments) fully explained the purpose and procedures of the study to each eligible family. The parents received a letter explaining the study, were given the opportunity to ask any questions about it, and signed a written informed consent form. Permission for children/adolescent’s participation in the study was required from both parents unless one was deceased, unknown, or legally incompetent. Besides written parental consent, the research assistant also obtained assent from children aged 8 years or more.

Then, a standardized comprehensive assessment of each family was performed, according to a written protocol that was distributed to all research assistants to ensure standardization of the assessment and data collection. The parents were asked to complete a number of self-completed measures separately without consulting each other. The research assistant carefully observed the patient’s behaviour, administered the assessment instruments, interviewed the parents, and reviewed the clinical chart together with the clinician caring for the patient.

### 2.4 Assessment Instruments

#### 2.4.1 Children/Adolescents

A structured form with close-ended questions specifically developed for the study was used to collect socio-demographic and clinical information, including demographic characteristics, medical history, information on current and previous medication and other forms of treatment (*e.g*., psychotherapy, occupational therapy, or speech therapy).

All children/adolescents were rated on the Global Impression item of the Clinical Global Impression scale – Severity (CGI-S) of illness (ranging from 1 = normal to 7 = extremely ill) [62], which provides an overall clinician-determined summary measure that takes into account all available information. Over the past four decades, the CGI has been shown to correlate well with several well-known rating scales across a wide range of psychiatric conditions [63].

The Children’s Global Assessment Scale (CGAS) [64] was administered to evaluate the social and psychological global functioning of all children/adolescents. The CGAS uses a range of scores from 1 (in need of constant supervision) to 100 (superior functioning) and has anchors at 10-point intervals including descriptions of psychopathology and functioning for each interval. A cut-off value of 60 or lower is indicative of definite impairment. The CGAS has been extensively used in research for more than two decades and is very similar to Axis V of the Diagnostic and Statistical Manual of Mental Disorders taxonomy. A recent study on Italian children with ASD provided evidence of convergent validity and reliability for the CGAS [65].

The cognitive abilities of all children (5-12 years old) were tested using Raven’s Coloured Progressive Matrices (RCPM) [66], a standardized test to measure non-verbal intellectual capacity in children. The RCPM consists of 36 items, presented in three sets of 12, which become progressively more difficult. Each item contains a pattern problem with one part removed and six pictured potential inserts, one of which contains the correct pattern. The number of correct answers is transformed into a non-verbal IQ score based on age-dependent normative data. The RCPM has good concurrent [67] and predictive validity [68] as well as split-half reliability [69]. In Italy, the RCPM has been standardized several times on the developmental Italian population [70].

For adolescents (aged 13-17 years old), the standard version of the Raven's Progressive Matrices (RPM) was used. The RPM demonstrated good internal consistency reliability, as well as content and convergent validity [71]. Both the RPM and RCPM are easy to administer and score, and they have been used extensively to assess the fluid-like component of intelligence of clinical populations of children [72].

In all children and adolescents, adaptive functioning was measured with the Italian version [73] of the Vineland Adaptive Behavioural Scales (VABS) [74]. This instrument has good psychometric properties, with demonstrable reliability and validity both for individuals who are developing typically and those with disabilities [74].

The VABS is a semi-structured parental interview that evaluates children’s ability to perform the daily activities required for personal and social sufficiency in four domains, *i.e*., Communication, Daily Living Skills, Socialization, and Motor Skills. The latter domain is measured only with children under 6 years old and older children and adults suspected of deficiencies in this area. Higher scores indicate greater adaptive functioning.

Raw scores and age-equivalent scores on each domain were calculated for all children, whereas we did not use standard scores because the Italian standardization of the instrument enables the comparison between children with typical and atypical development only by means of age-equivalent scores [75]. Given that this study included children up to 17 years old and only a minority of participants were rated on the Motor Skill domain, the VABS composite score was obtained by summing up the Communication, Daily Living Skills, and Socialization domain scores in order to have a composite score for all participants.

All participants who were clinically diagnosed with an ASD and their parents were administered the Italian version [76] of the Autism Diagnostic Observation Schedule (ADOS) [77] to confirm the diagnosis. The ADOS is a standardized, semi-structured assessment of social interaction, communication, play and imagination, and repetitive behaviours and interests. The developmental and language levels (*i.e*., nonverbal children, children with phrase speech, children and adolescents with fluent speech, adults with fluent speech) determine which one of four modules is administered. The ADOS has sound interrater reliability within domains and high internal consistency reliability, and it differentiates well between individuals with autism and those with other developmental disabilities [78].

The Childhood Autism Rating Scale (CARS) [79, 80] was used to evaluate the severity of symptoms of children with ASD, and to rule out a diagnosis of autism in children with DS. The CARS consists of 15 items, scored from 1 (age-appropriate behaviour) to 4 (severely autistic behaviour), that cover a broad range of behaviours that are impaired in autism (relating to people; emotional response; imitation; body use; object use; listening response; fear or nervousness; verbal communication; non-verbal communication; activity level; level and reliability of intellectual response; adaptation to change; visual response; taste, smell and touch response; and general impressions). The commonly accepted cut-off for autism is 30. Several studies reported high internal consistency, inter-rater and test–retest reliability, and criterion-related validity for the CARS [81].

#### 2.4.2 Parents

Parents were asked to complete a number of self-completed measures, which are described below.

The Family Problems Questionnaire (FPQ) [82] is a modified version of a self-completed questionnaire developed by the Italian National Institute of Health in collaboration with the Department of Psychiatry of the University of Naples. It consists of 34 items, grouped on the basis of factor analysis into five subscales assessing the caregiver’s (a) objective burden (*e.g*., constraints in social and leisure activities, difficulties going on holiday, work problems, economic difficulties, waking up at night, difficulties caring for other family members); (b) subjective burden (*e.g*., feelings of loss, sadness, guilt, worries about the future, embarrassment in public places, feeling unable to bear the situation much longer); (c) support received from professionals and members of the social network; (d) positive attitude toward the patient; (e) criticism of patient’s behaviour. Also, the instrument contains additional sections on economic costs, adverse impact on other children under age 12 years, and adverse impact on parental work. The FPQ has been validated in five languages (English, Italian, Portuguese, Greek, and German) [83]. The reliability and construct validity of the Italian version were established among key relatives of patients with schizophrenia and key relatives of patients with physical diseases [7]. The FPQ was administered in its entirety; however, given that the (d) and (e) subscales are less reliable than the others, that previous studies using this instrument have focused on the subscales assessing objective and subjective burden, and that social support was measured by a more specific and widely used instrument, only the data pertaining to the two burden subscales and the three additional sections were analyzed and reported here. In the mothers who took part in this study, the reliability of the Objective Burden scale as measured by coefficient Alpha was 0.88 (0.80, 0.83, 0.90 in the T1DM, DS, and ASD groups, respectively) and the reliability of the Subjective Burden scale was 0.81 (0.63, 0.83, 0.84 in the T1DM, ASD, and DS groups, respectively). Similar reliability figures were observed in fathers.

The 12-item version of the General Health Questionnaire (GHQ-12) [84] is a self-administered instrument designed to detect non-psychotic psychiatric morbidity and to measure depressive and anxiety symptoms. It has been translated into a variety of languages and has been widely used to detect non-psychotic psychiatric disorders in the community and general practice. It consists of 12 items, each rated on a 4-point frequency scale ranging from 1 to 4. Studies on primary care patients [85] and dermatological patients [86, 87] corroborated the validity and reliability of the Italian version of the GHQ-12. The scores were computed in a conventional way, collapsing adjacent responses to obtain a dichotomous scoring (0-0-1-1). We selected 3/4 as the cut-off threshold for psychiatric case identification because two previous Italian studies, which tested the GHQ-12 against standardized diagnostic interviews in general practice [85] and dermatological [87] settings, suggested that this cut-off threshold provides the best balance between sensitivity and specificity, and increases positive predictive value as much as possible while still retaining an acceptable level of sensitivity. The literature suggests that a sizable proportion of GHQ-12 high scorers have a psychiatric condition, usually a depressive disorder or an anxiety disorder, while others are experiencing substantial emotional distress and affective symptoms without meeting the full criteria for a psychiatric disorder.

The Brief COPE [88] is the abridged version of the Coping Orientation to Problems Experienced (COPE) inventory [89]. It is a theoretically-constructed, multidimensional coping scale with 14 subscales (acceptance, active coping, planning, humour, positive reframing, turning to religion, using emotional support, using instrumental support, behavioural disengagement, denial, self-blame, substance use, self-distraction, and venting of emotions), each consisting of two items that focus on distinct aspects of coping. The items are scored on a 4-point frequency scale and are summed to produce scale scores, with higher scores reflecting greater use of a particular coping strategy. Rather than using approaches described in the literature to summarize subscale scores into ‘problem-focused’ or ‘emotion-focused’ coping, which is a dichotomization of coping that may oversimplify the way people respond to stress [89], we grouped the subscales into four dimensions that were empirically identified in parents of children with ASD [52]. Although no single way to group coping responses fully captures the structure of coping [90], these dimensions may provide a valuable description of the structure of parental coping strategies in these families. The dimensions are Engagement (active coping, planning, using instrumental support, and use of emotional support), Disengagement (behavioural disengagement, substance use, and denial), Distraction (self-distraction, humour, self-blame, and venting of emotions), and Cognitive Reframing (positive reframing, acceptance, and turning to religion). This scoring method has been used in some previous studies on parents of children with ASD [18, 28, 52]. For each respondent, a score on each of the four coping dimensions was obtained by summing scores on the relevant Brief COPE subscales. In the mothers who took part in this study, the reliability of the Engagement, Disengagement, Distraction, and Cognitive Reframing dimensions as measured by coefficient Alpha was 0.80, 0.62, 0.71, and 0.63, respectively. Similar reliability figures were observed in fathers.

The Multidimensional Scale of Perceived Social Support (MSPSS) [91] is a 12-item self-completed questionnaire designed to measure the perceived social support from the significant other, the family, and friends. The instrument consists of three subscales, each composed of 4 items rated on a 7-point Likert scale. The total score is obtained by summing all subscale scores and ranges from 12 to 84. Higher scores indicate greater perceived social support. The Italian version of the instrument has been used in many previous studies [92-95].

The DRS-15 is a short, 15-item version of the Dispositional Resilience Scale [96], which is a self-report questionnaire developed to measure the construct of psychological hardiness. Individuals high in hardiness have a strong sense of life and work commitment, a greater feeling of control, and are more open to change and challenges in life. They tend to interpret stressful experiences as a normal aspect of existence, as a part of life that is overall interesting and worthwhile. Higher scores on the measure indicate greater psychological hardiness. The DRS-15 displayed good reliability and showed appropriate criterion-related and predictive validity in several samples, with respect both to health and performance under high-stress conditions [97, 98]. A recent study provided evidence of both validity and reliability for the Italian version of the DRS-15 [99].

The WHO Quality Of Life Spirituality, Religion and Personal Beliefs facet subscale (WHOQOL SRPB) is a subset of 4 items from the WHOQOL-100 [100], which is a comprehensive questionnaire developed by the World Health Organization to assess the dimensions of quality of life considered as the most important across different cultures and disease conditions [101]. The SRPB domain covers issues related to an individual's perception of quality of life in terms of how much spirituality, religion, and personal principles have a positive influence on the individual’s sense of meaning and purpose in life. Each item is rated on a 5-point Likert scale, with higher scores indicating better quality of life. The Italian version of the WHOQOL-100 has been thoroughly validated [102].

### 2.5 Statistical Analysis

All statistical analyses, except for structural equation modeling, were performed using SPSS for Windows, version 22.0 (SPSS Inc, Chicago, IL). All tests were two-tailed, with alpha set at 5%. The first set of analyses was carried out on all participants. First, demographic and clinical characteristics were summarised using appropriate descriptive statistics. Then, the Chi-square test and analysis of variance with Tukey-corrected post-hoc comparisons were used to test for differences between groups in categorical and continuous variables, respectively. In contingency tables, adjusted standardized residuals were calculated in order to identify cells in which the discrepancy between the observed and the expected frequency exceeded 1.96 and was therefore significant at p<0.05. In children, between-group comparisons were adjusted for age and sex, while in parents they were adjusted for age and for child’s age and sex.

Finally, multi-group structural equation modeling was used to examine how children’s and parents’ characteristics interact in influencing burden. These analyses were carried out using the M*plus* 7.11 statistical program [103]. The specific questions of interest were (1) whether child’s (global clinical severity, global functioning, and adaptive functioning, as measured by the CGI-S, C-GAS, and VABS composite score, respectively) and parent’s (hardiness, spiritual well-being, coping strategies, and perceived social support, as measured by the DRS-15, WHOQOL-100 SRPB, Brief COPE, and MSPSS, respectively) characteristics were related to burden; and (2) whether coping strategies and perceived social support mediated the effects of the other variables on burden.

Accordingly, in a first set of models we posited engagement, disengagement, distraction and cognitive reframing coping styles, along with perceived social support as a set of mediators, indirectly connecting a set of predictors (global clinical severity, global functioning, adaptive functioning, hardiness, and spiritual well being) on objective and subjective family burden. According to recommended standards, we (1) posited the predictors correlated among them, (2) the mediators as correlated among them, and (3) tested and included direct effects of the predictors on the outcomes (*i.e*., subjective and objective burden) if statistically significant [104]. We tested separate models for mothers and fathers, and for objective and subjective burdens. However, we fitted the above models simultaneously on the three different groups of children with ASD, T1DM, and DS.

In the second set of models, we repeated the above models only in the ASD group, by including the severity of intellectual disability, which could not be entered in the multi-group models as its variance in the T1DM group was zero, and by replacing the variable global clinical severity with severity of autism symptoms as measured by the CARS.

In all models, we included parent’s age and child’s sex and age as covariates, by regressing all variables included in the models on them.

All variables were posited in the model as single indicators with fixed residual variance.

Whereas there has been considerable debate in the literature concerning the use of maximum likelihood estimation (ML) with ordinally-scaled variables treated as continuous [105], different simulation studies have found that ML performs well with variables with four or more categories [106] and under less-than-optimal analytical conditions (for example, in the presence of small sample sizes and moderate departures from normality). However, because multivariate normality was non-tenable in the present sample [multivariate skewness and kurtosis coefficients ranged from 120.77 (*p* < .05) to_88.81 (*p* < .05) and were significant], we employed the Satorra-Bentler [107] scaled chi-square statistic (SBχ^2^) and standard error, which takes into account the non-normal distribution of the data (M*plus* estimator = MLM: Maximum Likelihood estimation with Satorra-Bentler corrections). As a sensitivity test, we also ran some of the models using the WLS estimator. As the parameter estimates were nearly identical, we present the results obtained using the MLM estimator.

Because the chi-square is highly sensitive to sample size, the SB*χ^2^* likelihood ratio statistic was supplemented with other indices of model fit, such as the Comparative Fit Index (CFI) and the Root Mean Square Error of Approximation (RMSEA) with associated 95% confidence interval. We accepted CFI values greater than .95 and RMSEA values lower than .08 [108].

To investigate mediation, we used the asymmetric confidence interval method to formally test the significance of indirect effects [104]. The critical values for the upper and lower confidence limits for indirect effects were tested by using the Monte Carlo Method for Assessing Mediation CI method [109] with 20,000 replications.

To be parsimonious and increase the subject/parameters ratio, we maintained in the model only significant paths (*i.e*., *p*< .05), and constrained to zero non-significant paths if this did not decrease model fit (both for main variables and covariates), In doing so, we followed a “one-step” procedure aimed at decreasing the risk of capitalizing on chance. First, we estimated the model; then, we determined which parameters were above the threshold of statistical significance (*p* = .05); finally, in a single step, we fixed all parameters above this threshold to zero. As the difference between two scaled chi-squares for nested models is not distributed as a chi-square, the tenability of the constraints imposed for testing measurement invariance was examined with the scaled difference chi-square (SB-DCHI) [110].

## 3 RESULTS

### 3.1 Characteristics of Participants

Overall, a total of 644 mothers and 536 fathers, belonging to 659 families, were involved in the study. On average, they were in their forties and were well educated, as most of them had senior high school education or higher; the majority of parents were employed in paid work, and only a few were unmarried, separated, or divorced. For 521 families (280 ASD, 110 DS, 131 T1DM), both parents took part in the study, while for 123 (71 ASD, 30 DS, 22 T1DM) and 15 (8 ASD, 5 DS, 2 T1DM) families only the mother or father was involved, respectively. Parents’ sociodemographic characteristics are detailed in Table **1**.

A total of 659 children took part in the study; 359 were affected by ASD, 145 by DS and 155 by T1DM. As expected given the epidemiology of ASD, males were markedly over-represented in the ASD group, while the gender distribution was more balanced in the T1DM group and especially the DS group. Most children had one or more siblings, and only very few lived in a single-parent family. Patients’ sociodemographic and clinical characteristics are shown in detail in Tables **2** and **3**, respectively.

### 3.2 Parental Burden and Depressive and Anxiety Symptoms

#### 3.2.1 Between-Group Comparisons

Table **4** summarizes the mean scores on the FPQ and GHQ-12 by group. The FPQ was completed by all fathers and by all mothers, except two (99.7%). The parents of children with ASD reported significantly higher levels of both objective and subjective burden than the parents of children with DS or T1DM. Parents of children with ASD displayed higher scores on all the items pertaining to these scales, which suggests that their greater burden was not linked to specific parenting experiences. Also, the adverse impact on other children and parental work was significantly greater in the families with a child affected by ASD than in the families with a child affected by DS or T1DM. Moreover, child health-related expenses were markedly higher in the families with a child affected by ASD than in the families with a child affected by T1DM and, to a less marked but still significant extent, the families with a child affected by DS.

All except a few parents (630 mothers, 97.8%; 526 fathers, 98.1%) completed the GHQ-12. Significant depressive and anxiety symptoms were found in a high proportion of parents of children with ASD, as 113 (33.0%) of 342 mothers and 85 (29.9%) of 284 fathers scored above the threshold for probable psychiatric caseness on the GHQ-12. As compared with the ASD group, the proportion of participants with significant depressive and anxiety symptoms was lower among mothers (29 of 138; 21.0%) and fathers (16 of 112; 14.3%) of children with DS. The difference is significant for both mothers and fathers after adjustment for age and child’s age but loses significance in mothers after adjusting also for child’s sex, due to the absence of between-group differences in mothers of female children. The parents of children with T1DM occupied an intermediate position (40 of 150 mothers; 26.7%; 31 of 130 fathers; 23.8%) with no significant differences from the two other groups.

#### 3.2.2 Correlation and Comparison Between Mothers and Fathers in the Families where Both Parents Took Part in the Study

In the families where both parents took part in the study, the ratings of both subjective and objective burden were found to be moderately to strongly correlated between mothers and fathers (r=0.62 and 0.70, respectively, in families of children with ASD; r=0.64 and 0.69 in families of children with DS; r=0.51 and 0.46 in families of children with T1DM; all p<0.001). The severity of symptoms of depression and anxiety as expressed by the GHQ-12 total score, too, showed a significant, though lower, correlation between mothers and fathers (r=0.33, p<0.001; r=0.28, p<0.01; r=0.31, p<0.01 in families of children with ASD, DS, and T1DM, respectively).

Regarding the between-gender comparison, subjective burden was found to be significantly higher in mothers as compared with fathers in families of children with ASD (mean score 13.73±4.54* vs. *12.76±4.11; p=0.008), while the difference fell short of statistical significance in families of children with DS (11.15±3.58* vs. *10.50±3.08; p=0.15) and T1DM (11.28±2.92* vs. *10.66±2.50; p=0.065). Objective burden did not significantly differ between mothers and fathers either in families of children with ASD (mean score 14.64±5.41* vs. *13.92±5.42; p=0.11) or with DS (12.75±4.70* vs. *12.15±4.21; p=0.32) or T1DM (11.89±3.47* vs. *11.18±2.71; p=0.066). Mothers and fathers did not differ with regard to the severity of depressive and anxiety symptoms, as in all parent groups the proportion of mothers scoring above the threshold for probable psychiatric caseness on the GHQ-12 did not significantly differ from the proportion of fathers (all p>0.15) and there was no significant difference between mothers and fathers in GHQ-12 mean score (all p>0.15).

### 3.3 Coping Resources and Strategies

Table **5** summarizes the mean scores on the Brief COPE, MSPSS, DRS-15 and WHOQOL-100 SRPB by group. As compared with the parents of children with T1DM, both the mothers and fathers of children with ASD, as well as the mothers of children with DS, reported the significantly more frequent use of engagement and cognitive reframing coping strategies. On the other hand, as compared with the mothers of children with T1DM, the mothers of children with ASD reported greater use of distraction coping. Finally, the fathers of children with T1DM reported greater use of disengagement coping than the fathers of children with DS.

Overall, the parents of children with ASD reported reduced levels of perceived social support. As compared with the mothers of children with DS and T1DM, the mothers of children with ASD scored significantly lower on the MSPSS. Also, the fathers of children with ASD scored significantly lower on the MSPSS than the fathers of children with T1DM. The MSPSS mean scores of the parents of children with ASD were also several points lower than the mean scores we observed in previous studies on healthy nurses [111] and patients with mild skin diseases [94]. Inspection of the subscales suggested that the parents of children with ASD perceived lack of support from family and friends, rather than from the spouse. In fact, the mothers of children with ASD perceived significantly less support from family and friends as compared with the mothers of children with DS and T1DM, and the fathers of children with ASD perceived significantly less support from family and friends as compared with the fathers of children with T1DM. On the other hand, the only between-group difference in mean scores on the MSPSS Significant Other subscale was a significantly lower score in mothers of children with ASD as compared with the mothers of children with DS.

All groups reported mean levels of spiritual well-being, as measured by the SRPB domain of the WHOQOL-100, which compared favourably with those we observed in a previous study on patients with temporal lobe epilepsy [112]. No between-group differences were observed.

With regard to hardiness, the three groups displayed similar scores on the DRS-15; these scores were also commensurate with those observed in non-clinical subjects of comparable age in the validation study of the Italian version of the instrument [99].

### 3.4 Structural Equation Modeling

The mediational model was fitted in a single step in all groups simultaneously, as a multiple-group structural equation modeling, separately for type of burden (*i.e*., subjective or objective) and observer (*i.e*., mother or father). Below, we describe the major results separately for type of burden and observer.

#### 3.4.1 Objective Burden

##### 3.4.1.1 Mothers

The multiple group mediational model for mothers fitted the data well: *χ^2^*(172) = 191.27, *p* = .15, CFI = .99, TLI = .98, RMSEA = .023 (95%CI = .00, .039), SRMR = .059. Fig. (**1**), Panel A presents model estimates obtained in the mothers of children with ASD. The objective burden was significantly and positively predicted by engagement and disengagement coping, and negatively predicted by social support that was the stronger direct predictor. In turn, engagement coping was significantly and negatively predicted by hardiness and significantly and negatively predicted by the child’s adaptive functioning; disengagement coping by spiritual wellbeing; distraction and cognitive reframing coping; perceived social support by hardiness and spiritual wellbeing. There were significant total indirect effects on objective burden of: (1) hardiness trough engagement coping (.039; 95%CI = .016, .069) and perceived social support (-.11; 95%CI = -.172, -.062); (2) of spiritual wellbeing through disengagement coping (-.04, 95%CI =-07, -.006), and through social support (-.06, 95%CI =-.094, -.016). The indirect effect of adaptive functioning on objective burden through engagement coping was instead not significant (-.002, 95%CI = -.005, .00). Significant covariate effects were those of maternal age on disengagement coping (.11, *p* = .017), of child gender on reframing coping (.12, *p* = .007), and of child’s age on global clinical severity (.39, *p*< .001), and global functioning (-.31, *p*< .001).

Results for mothers of children with T1DM are presented in Fig. (**1**), Panel C. As it can be seen, only a direct effect of perceived social support on the objective burden, and of global clinical severity on engagement coping were found. No indirect effects were found. Significant covariate effects were those of maternal age (.22, *p* = .007) and child’s age (-.43, *p*< .001) on objective burden, of maternal age (.16, *p* = .050) on global functioning, and of child’s age (.80, *p*< .001) on adaptive functioning.

Results for mothers of children with DS are presented in Fig. (**1**), Panel E. In this case, we found a significant direct and positive path linking engagement coping to objective burden, and a negative direct path linking perceived social support to objective burden. Moreover, there were significant positive predictions of engagement coping with hardiness and spiritual wellbeing, and a positive prediction of distraction coping by hardiness. Cognitive reframing coping was significantly and positively predicted by global clinical severity and spiritual wellbeing, but negatively predicted by global functioning; finally, perceived social support was significantly and positively predicted by hardiness. There were significant indirect effects on objective burden of (1) hardiness on burden trough engagement coping (.026, 95%CI =.005, .071). The indirect effects of hardiness through engagement coping and of spiritual wellbeing on objective burden were instead not significant. The only significant covariate effect was that of child’s age (.57, *p*< .001) on adaptive functioning.

The structure of the mediational model was quite different across groups, as shown by the different pattern of significant paths. Nonetheless, there was an equivalent path across the three groups (*i.e*., the path linking perceived social support to objective burden), as indicated by a partial chi-square test: SB-DCHI = 7.55(2), p = .06. There were also three other paths that were analogous across the ASD and DS groups: (1) hardiness to engagement coping, (2) hardiness to distraction coping, (3) hardiness to perceived social support. Among the above paths, however, only the one linking hardiness to distraction coping was statistically equivalent across the two groups: SB-DCHI = 2.01(1), *p* = .17.

##### 3.4.1.2 Fathers

The multiple group mediational model for fathers fitted the data well: *χ^2^* (172) = 190.70, *p* = .17, CFI = .99, TLI = .98, RMSEA = .024 (95%CI = .00, .042), SRMR = .064. Fig. (**1**), Panel B presents the model estimates obtained in fathers of children with ASD. Objective burden was significantly and positively predicted by engagement and disengagement coping, and negatively and significantly predicted by adaptive functioning, and by perceived social support, which was the stronger direct predictor. Spiritual wellbeing significantly predicted engagement, disengagement, and cognitive reframing coping, and perceived social support. The latter was also significantly predicted by hardiness. There were significant indirect effects of (1) spiritual wellbeing through engagement (.034, 95%CI = .008, .061) and disengagement (-.033, 95%CI = -.061, -.005) coping strategies, and social support (-.053, 95%CI = -.103, -.003), and (2) of hardiness through social support (-.10, 95%CI = -.153, -.040). Significant covariate effects were those of paternal age (-.14, *p* = .009) on hardiness, and of child’s age on global clinical severity (.36, *p*< .001), global functioning (-.24, *p*< .001), and adaptive functioning (.12, *p* = .042).

Results for fathers of children with T1DM are presented in Fig. (**1**), Panel D. Objective burden was significantly predicted by engagement and disengagement coping. In turn, engagement coping was predicted by hardiness, disengagement coping by global functioning, cognitive reframing coping by hardiness, and perceived social support by spiritual wellbeing. The indirect effect of hardiness on burden though engagement coping (.05, 95%CI =-.003, .10) was not statistically significant. Significant covariate effects were those of child’s age (-.24, *p* = .003) on objective burden and adaptive functioning (.78, *p< .*001), and of gender on hardiness (.17, *p* = .043),

Finally, the mediational model for fathers of children with DS is presented in Fig. (**1**), Panel F. Objective burden was significantly predicted only by distraction coping. In turn, distraction coping was predicted by global functioning, cognitive reframing coping by spiritual wellbeing, and perceived social support by global clinical severity, hardiness, and spiritual wellbeing. The indirect effect of global functioning on objective burden through distraction coping was significant (-.02, 95%CI =-.036, -.004). Significant covariate effects were those of child’s age on disengagement coping (.20, *p* = .019), and on adaptive functioning (.57, *p* < .001).

The structure of the mediational model was quite different across groups, as shown by the different pattern of significant paths. Moreover, the only path that consistently emerged across groups (*i.e*., the one linking spiritual wellbeing to social support) differed significantly across groups (SB-DCHI = 21.55(2), p < .001). It emerged as statistically equivalent only across the ASD and the DS groups (SB-DCHI = 2.51(1), *p* = .11).

#### 3.4.2 Subjective Burden

##### 3.4.2.1 Mothers

The multiple group mediational model for mothers fitted the data well: *χ^2^*(166) = 155.61, *p* = .70, CFI = 1.00, TLI = 1.01, RMSEA = .00 (95%CI = .00, .025), SRMR = .050. Fig. (**2**), Panel A presents model estimates obtained in the mothers of children with ASD. Their subjective burden was significantly and positively predicted by global clinical severity and distraction coping, and negatively and significantly predicted by disengagement coping, perceived social support, hardiness, and spiritual wellbeing. In turn, engagement coping was positively predicted by adaptive functioning and negatively predicted by hardiness; disengagement coping was negatively predicted by spiritual wellbeing; social support and distraction and cognitive reframing coping were predicted by hardiness and spiritual wellbeing.

There were also significant and positive indirect effects of hardiness on objective burden trough distraction coping (.026, 95%CI =.009, .051), and negative indirect effects through perceived social support (-.05, 95%CI =-.085, -.028). Spiritual wellbeing revealed a negative indirect effect on objective burden trough disengagement coping (-.038, 95%CI =-.082, -.006), through distraction coping (-.043, 95%CI =-.079, -.014) and trough perceived social support (-.056, 95%CI =-.126, -.025). Significant covariate effects were those of maternal age on disengagement coping (.11, *p* = .019), gender on reframing coping (.12, *p* = .007), child’s age on global clinical severity (.39, *p* < .001) and global functioning (.31, *p* < .001).

Results for mothers of children with diabetes are presented in Fig. (**2**), Panel C. As it can be seen, we found only two direct negative predictions of subjective burden by perceived social support and global clinical severity. The latter significantly and positively predicted engagement coping. Significant covariate effects were those of child’s age on subjective burden (-.20, *p* = 007) and adaptive functioning (.80, *p* < .001), and of maternal age on global functioning (.16, *p* = .050).

Finally, results for mothers of children with Down syndrome were presented in Fig. (**2**), Panel E. In this case, we found significant and positive direct predictions of subjective burden by engagement and disengagement coping, and a negative prediction by spiritual wellbeing. Moreover, engagement coping was significantly and positively predicted by hardiness and spiritual wellbeing; distraction coping was significantly and positively predicted by global functioning and hardiness; Cognitive reframing coping was significantly and positively predicted by global clinical severity and spiritual wellbeing, but negatively and significantly predicted by global functioning; finally, perceived social support was significantly predicted by hardiness. There were significant and positive indirect effects of hardiness on objective burden through engagement coping (.044, 95%CI =.001, .098) and negative effects through perceived social support (-.030, 95%CI =-.059, -.005. Spiritual wellbeing revealed a positive indirect effect on objective burden through engagement coping (.081, 95%CI =.016, .15). There was a significant covariate effect of child’s age on adaptive functioning (.57, *p*< .001).

The structure of the mediational model was quite different across groups, as indicated by the different pattern of significant paths. Only one path consistently emerged across groups (*i.e*., the path linking perceived social support to subjective burden), and proved to be statistically equivalent SB-*∆χ^2^* = 3.98(2), *p* = .14. No other path was statistically equivalent across groups, except those predicting distraction and engagement coping by hardiness in the ASD and DS groups: SB-*∆χ^2^* = 4.10 (2) *p* = .13

##### 3.4.2.2 Fathers

The multiple group mediational model for fathers fitted the data well: *χ^2^* (173) = 206.33, *p* = .04, CFI = .97, TLI = .96, RMSEA = .033 (95%CI = .001, .049), SRMR = .067. Fig. (**1**), Panel B presents the model estimates obtained in the fathers of children with ASD. Subjective burden was significantly predicted by engagement and disengagement coping, perceived social support, and hardiness. Spiritual wellbeing significantly predicted perceived social support and all coping strategies except distraction. Perceived social support was also significantly predicted by hardiness. There were significant and positive indirect effects of (1) spiritual wellbeing on objective burden through engagement coping (.031, 95%CI =.005, .064), and negative indirect effects through disengagement coping (-.066, 95%CI =-.126, -.022) and perceived social support (-.048, 95%CI =-.101, .010); (2) hardiness had a negative indirect effect on subjective burden through social support (-.053, 95%CI =-.088, -.023). Significant covariate effects were those of paternal age on hardiness (-.14, *p* = .009), and of child's age on global clinical severity (.36, *p* < .001), global functioning (-.24, *p* < .001), and adaptive functioning (.12, *p* = .043).

Results for fathers of children with diabetes are presented in Fig. (**2**), Panel D. Subjective burden was significantly predicted only by cognitive reframing coping. In turn, engagement coping was predicted by hardiness; disengagement coping by global functioning; cognitive reframing coping by hardiness; and perceived social support by spiritual wellbeing. There was a significant and negative indirect effect of hardiness on subjective burden through cognitive reframing (-.10, 95%CI =-.18, -.020). There were significant covariate effects of gender on hardiness (.17, *p* = .041), and of child’s age on spiritual wellbeing (.21, *p* = .012) and adaptive functioning (.77, *p* < .001).

Finally, the mediational model for fathers of children with DS is presented in Fig. (**2**), Panel F. Objective burden was significantly predicted by distraction coping and hardiness. In turn, distraction coping was predicted by global functioning; cognitive reframing coping by spiritual wellbeing; and social support by hardiness and spiritual wellbeing. There was a significant and negative indirect effect of global functioning (-.01, 95%CI =-.026, -.000) on objective burden though distraction coping. There was a significant covariate effect of child’s age on adaptive functioning (.57, *p* < .001).

The structure of the mediational model was quite different across groups, as shown by the different pattern of significant paths. Only the path from spiritual wellbeing to cognitive reframing across the ASD and DS groups was statistically equivalent (SB-∆*χ^2^* = 2.42(1), *p* = .12) . No other path was statistically equivalent across groups.

#### 3.4.3 Specific Models for Parents of Children with ASD

For the ASD group, we re-ran all models by including the severity of intellectual disability and replacing global clinical severity with the severity of autism symptoms as measured by the CARS. Below, we briefly summarize the main findings. Covariate effects in these models were equal to those observed in the previous analogous model. No covariate effect was observed on child’s intellectual disability and severity of autism symptoms.

##### 3.4.3.1 Mothers

The mediational model for objective burden fitted the data well: *χ^2^* (59) = 131.58, *p* = <.01, CFI = .96, TLI = .92, RMSEA = .052 (95%CI = .040, .064), SRMR = .060. This model is shown in Fig. (**3**), Panel A. Mother’s objective burden was significantly and positively predicted by engagement and disengagement coping, child’s intellectual disability, and autism symptom severity; on the other hand, it was significantly and negatively predicted by the perceived social support. In turn, engagement coping was significantly positively predicted by autism symptom severity, while it was significantly negatively predicted by adaptive functioning and hardiness; disengagement coping was negatively predicted by spiritual wellbeing; distraction coping was significantly positively predicted by hardiness, whereas it was negatively and significantly predicted by spiritual wellbeing; cognitive reframing coping was significantly predicted by hardiness and spiritual wellbeing; perceived social support was significantly predicted by global functioning, hardiness, and spiritual wellbeing. There were significant negative indirect effects on objective burden of: (1) global functioning through perceived social support (-.045, 95% CI = -.075, -.015), (2) adaptive functioning through engagement coping (-.024, 95% CI = -.043, -.004); (3) hardiness through perceived social support (-.079, 95% CI = -.116, -.042); (4) spiritual wellbeing through disengagement (-.023, 95% CI = -.044, -.001) and through perceived social support (-.074, 95% CI = -.043, -.004). Instead, hardiness revealed a significant but positive indirect effect on objective burden through engagement (.043, 95%CI = .019, .067).

The fit of the mediational model for subjective burden was acceptable: *χ^2^*(59) = 97.59, *p* = <.01, CFI = .98, TLI = .96, RMSEA = .038 (95%CI = .024, .051), SRMR = .056. This model is shown in Fig. (**3**), Panel C. Mother’s subjective burden was significantly and positively predicted by disengagement and distraction coping, and by autism symptom severity. Instead, it was negatively and significantly predicted by global functioning, spiritual wellbeing, and perceived social support. In turn, engagement coping was positively and significantly predicted by hardiness and autism symptom severity; disengagement coping was negatively and significantly predicted by spiritual wellbeing; distraction coping was positively and significantly predicted by hardiness, and negatively by spiritual wellbeing; cognitive reframing coping was positively and significantly predicted by hardiness and spiritual wellbeing; and perceived social support was positively and significantly predicted by global functioning, hardiness, and spiritual wellbeing. There were also significant and negative indirect effects on burden of global functioning through perceived social support (-.008, 95%CI =-.013, -.003); of hardiness trough social support (-.046, 95%CI = -.072, -0.025); and of spiritual wellbeing through disengagement (-.026, 95%CI =-.059, -.002), distraction coping (-.026, 95%CI =-.050, -.006), and social support (-.070, 95%CI =-.121, -.032). Hardiness, instead, revealed a significant and positive indirect effect on subjective burden trough distraction coping (.020, 95%CI =.006, .039).

##### 3.4.3.2 Fathers

The mediational model for objective burden fitted the data well: *χ^2^* (56) = 57.64, *p* =.41, CFI = 1.00, TLI = 1.00, RMSEA = .011 (95%CI = .00, .041), SRMR = .048. This model is shown in Fig. (**3**), Panel B. Objective burden was significantly and positively predicted by engagement and disengagement coping, autism symptom severity, and intellectual disability; on the other hand, it was significantly and negatively predicted by the perceived social support. Spiritual wellbeing significantly negatively predicted engagement and disengagement coping, while it significantly positively predicted cognitive reframing coping. Social support was significantly and positively predicted by hardiness and spiritual wellbeing.

There were significant and negative indirect effects of hardiness on objective burden through perceived social support (-.110, 95%CI =-.170, -.060), and of spiritual well-being through engagement (-.057, 95%CI =-.105, -.016) and disengagement coping (-.073, 95%CI =-.134, -.023), and through perceived social support (-.085, 95%CI =-.185, -.014).

The model for subjective burden fitted the data well: *χ^2^* (56) = 62.83, *p*< .001, CFI = .99, TLI = .98, RMSEA = .022 (95%CI = .000, .046), SRMR = .050. This model is presented in Fig. (**3**), Panel D. There were direct positive and significant paths from disengagement coping and autism symptom severity to the subjective burden. Perceived social support, instead, significantly negatively predicted subjective burden. Spiritual wellbeing significantly and positively predicted perceived social support, engagement coping, and cognitive reframing coping, while it significantly negatively predicted disengagement coping. Hardiness significantly positively predicted perceived social support. There were significant and negative indirect effects on subjective burden of hardiness (-.055, 95%CI =-.092, -.023) through social support, and of spiritual wellbeing through disengagement coping (-.070, 95%CI =-.14, -.021) and perceived social support (-.040, 95%CI =-.092, -.023).

## 4 DISCUSSION

### 4.1 Comparison of Burden Between Families

Consistently with previous literature [5, 8], this study showed that the parents of children with ASD carry a severe burden of care and frequently suffer from significant depressive and anxiety symptoms.

The parents of children and adolescents with ASD reported significantly higher levels of both objective and subjective burden than the parents of children and adolescents with DS or T1DM. The mean level of objective burden observed in the parents of children with ASD was higher than the level reported by parents (mothers, for the most part) of children, adolescents, and young adults affected by muscular dystrophies [113], and was comparable to the level observed in the relatives (mothers, for the most part) of adult patients with schizophrenia in five European countries [83, 114]. With regard to parental subjective burden, the mean levels observed in ASD were similar to those observed in muscular dystrophies and slightly lower than those found in schizophrenia.

Also, specific aspects of burden such as child health-related expenses and the adverse impact on parental work and on other children were significantly greater in the families with a child affected by ASD than in the families with a child affected by DS or T1DM. Most previous studies, with few exceptions [115], have reported that parents of children with ASD endure substantial financial burdens, in the form of high out-of-pocket health care costs [22, 24, 25] and decreased workforce involvement [21-23].

Significant symptoms of depression and anxiety were also found to be quite common among parents of children with ASD, as almost one-third of them scored above the threshold for probable psychiatric morbidity on the GHQ-12. For mothers of male children and for fathers, the proportion of high scorers on the GHQ-12 was significantly higher in the ASD group than in the DS group, and was also higher than in the T1DM group, though the difference did not reach significance.

In agreement with our findings, previous studies on mothers and fathers of children with ASD [9, 10] and on mothers of toddlers [116], children [12], and adolescents with ASD [11] reported increased depressive symptoms and high prevalence of probable clinical depression or significant psychological distress. Also, studies comparing the parents of children with ASD with the parents of typically developing children [13, 15, 16, 18, 19, 117, 118] and of children with developmental delay [14], intellectual disability [17, 118] or DS [13, 16] consistently reported higher levels of depression, anxiety, emotional distress, and a variety of psychopathological dimensions in parents of children with ASD. It should be noted that increased anxiety and depressive symptoms among parents of children with ASD might not always be related to caregiving burden, as the literature suggests that the greater risk of psychopathology displayed by the parents of children with ASD [119] is only partly explained by parental stress and family burden [120, 121]. Together with previous literature, our findings draw attention to the high level of distress from depressive and anxiety symptoms suffered by the parents of children with ASD and suggest that this issue deserves careful clinical attention.

### 4.2 Comparison Between Mothers and Fathers

A distinctive feature of this study is that in most families both parents were involved. This is important because in Italy and numerous other countries many fathers are substantially involved in caring for their children. However, with very few exceptions, previous studies on parenting stress and burden in parents of children with ASD have included only mothers or mothers with a small minority of fathers. This creates a knowledge gap, as research carried out on mothers of children with ASD may not be generalizable to fathers, given common differences between mothers’ and fathers’ domestic roles.

Previous research carried out to date suggests that both in western and eastern countries the mothers of children with ASD suffer from increased levels of stress [122-126], anxiety, and depression [127] compared with fathers. However, fathers suffer, too, and in a Canadian study, they reported even higher levels of stress than mothers [37].

In this study, we observed in all three groups a significant correlation between mothers’ and fathers’ ratings of subjective and objective burden, as well as of depressive and anxiety symptoms. The correlation was particularly strong for the ratings of burden, which corroborates the validity of the assessment of this key variable and suggests that mothers and fathers share a similar view of the challenges and difficulties faced by the family.

Only few mother-father differences in parental burden were identified. In the families with a child affected by ASD, the mothers reported greater subjective burden than fathers, while no difference between mothers and fathers was found for objective burden and depressive and anxiety symptoms. In the other two groups, no differences between mothers and fathers were observed in subjective and objective burden and in depressive and anxiety symptoms. This pattern of findings suggests that, overall, the mothers and fathers of children with ASD, DS, and T1DM experience similar levels of burden, but that mothers may more often feel overwhelmed when facing the particularly heavy caregiving demands associated with parenting a child with ASD.

### 4.3 Comparison of Coping Resources and Strategies Between Families

Previous studies of coping resources other than social support among parents of children with ASD have been scarce. Differences in spiritual wellbeing between parents of children with ASD and other parent groups were not previously investigated. Hardiness was examined in one study, which found that mothers of children with ASD reported less hardiness than mothers of children with intellectual disabilities or typically developing children [60]. Some studies examined differences in other constructs that share similarities with hardiness, such as sense of coherence (SOC) and internal locus of control. People with strong SOC perceive the world as sensible, ordered, predictable, manageable and meaningful, and view problems as important challenges worth facing. In previous studies, mothers of children with ASD were found to have lower levels of SOC than parents of children with non-autistic intellectual disabilities [117] and parents of typically developing children [117, 128, 129]. The same finding was observed in fathers, too, with one exception [117]. Similarly, internal locus of control, *i.e*., the perceived personal controllability of life events, was found to be lower in parents of children with ASD than in parents of children with DS and of children with no developmental disorder [16].

In our study, the mothers and fathers of children with ASD displayed levels of hardiness and spiritual well-being that were similar to those observed in the parents of children with DS and T1DM, which suggests that the parents of children with ASD do not lack personal coping resources and are remarkably resilient.

Also, our findings, overall, do not suggest that the parents of children with ASD are more likely to use coping strategies commonly regarded as less beneficial for wellbeing in the long term, such as passive or avoidant emotion-focused coping, or less likely to use strategies assumed to be more beneficial, such as active, problem-focused coping. In fact, both mothers and fathers of children with ASD reported greater use of active, problem-focused strategies such as engagement coping than the parents of children with T1DM. They also reported greater use of the supposedly beneficial cognitive reframing coping. Also, they did not differ in the use of disengagement coping from the other parent groups. The only finding suggesting greater use by them of a strategy assumed to be less beneficial was the observation of a greater use of distraction coping in the mothers of children with ASD as compared with the other groups.

This finding is consistent with the previous literature. Among persons with a child diagnosed with ASD, the coping scores were found to be similar to the norms for the instrument used [130]. In a Lebanese study, the parents of children with ASD were found to use a variety of adaptive coping strategies. While they reported using disengagement coping strategies more frequently than mothers of typically developing children, they also reported greater use of engagement coping strategies and similar use of distraction and cognitive reframing coping [18]. Another study revealed many similarities in coping strategies between parents of children with ASD and parents of typically developing children [128], as the only difference observed was that parents of children with ASD employed escape-avoidance coping more frequently. Therefore, consistently with previous studies, our findings suggest that the use of coping strategies by parents of children with ASD is as functional as that by parents of children with other health conditions.

The finding of presumably functional coping in combination with average levels of hardiness and spiritual wellbeing, in the context of severe caregiving burden, creates a picture that resembles the ‘resilient disruption’ model of family adaptation developed to account for the impressive resilience of many families to the stress associated with disability, which posits that families are both disrupted by and resilient to the stress associated with raising a child with disability [131].

On the other hand, social support was found to be a critical issue for the parents of children with ASD. The pattern of findings suggests that it was support from family and friends, rather than from the spouse, to be perceived as poor by parents of children with ASD. In fact, they reported reduced levels of perceived social support from family and friends, both in absolute terms and relative to the other groups.

This finding of low social support from the extended family and friends is consistent with those of qualitative studies, in which many parents of children with ASD stated that family members did not understand the disorder, even if they accepted the diagnosis, and thus could not provide the needed support. Isolation was a common experience, as friends avoided parents, thus leaving them with a sense of diminished support [132, 133].

### 4.4 Relationships Between Child and Parent Characteristics and Parental Burden

We used structural equation modeling to investigate the relations between parental burden and a number of child and parent characteristics deemed as relevant based on previous research and theoretical considerations. We tested the direct effect of all variables on the parental burden and the possible mediating role of social support and coping strategies.

As far as the parents of children with ASD are concerned, given the close similarity in findings between the models obtained in the multi-group and single-group analyses, we discuss the latter models, which are more informative as they included intellectual disability and autism symptom severity as measured by the CARS. The findings of multi-group models are discussed with respect to the issue of whether the processes active in the families of children with ASD operated in similar ways in the other two groups.

#### 4.4.1 Child characteristics and burden.

##### 4.4.1.1 Demographic Variables

Some previous studies suggested a negative relationship between child’s age and maternal burden, with mothers of older children with ASD reporting lower stress levels [27-29]. However, most previous studies found no age-related effects [42-44]. Consistently with the bulk of the literature, we did not find any association between objective or subjective burden and the child’s demographic variables, either in mothers or in fathers.

##### 4.4.1.2 Global and Adaptive Functioning

In mothers of children with ASD, the child’s global and adaptive functioning displayed a negative indirect effect on objective burden through perceived social support and through engagement coping, respectively. The child’s global functioning also displayed a direct negative association with the subjective burden and had also a negative indirect effect on subjective burden through perceived social support.

These findings are in agreement with most of the literature. All previous studies, with only one exception [42] reported that child’s adaptive behaviour and skills [31, 37-39] are associated with parental stress or psychological distress. Our findings also extend previous observations on the subjective aspects of burden to the objective burden. The pattern of indirect effects suggests that the parents of the children with a greater functional impairment may have more difficulties in maintaining existing social bonds or creating new ones, thus leading to increased burden through a loss of social support.

##### 4.4.1.3 Intellectual Disability

Previous studies examining the association between the child’s intellectual disability and parental burden provided inconsistent findings. On one hand, a number of studies reported an association between child’s cognitive abilities [37, 40, 41], and parental stress or psychological distress. On the other hand, some studies reported either a lack of association between child IQ and parental stress [31] or the absence of a unique contribution of cognitive deficits to variance in parenting stress when considered together with other child characteristics [9].

Our findings concerning subjective burden are in agreement with the previous negative studies, as the severity of the child’s intellectual disability was not associated with subjective burden. However, in both mothers and fathers of children with ASD, the severity of the child’s intellectual disability was directly associated with increased objective burden. This pattern of findings suggests that although the parents of intellectually disabled children with ASD face greater practical problems, the influence of intellectual disability on their emotional wellbeing is relatively mild when compared with the impact of autistic symptoms and impaired adaptive functioning, which seem to be the main child-related sources of parental subjective burden.

##### 4.4.1.4 Severity of ASD Symptoms

The most consistent predictor of burden was the severity of autistic symptoms, that was directly associated with objective and subjective burden in both mothers and fathers of children with ASD. The use of the CARS as a measure of severity suggests that the association between ASD severity and parental burden cannot be explained by information bias.

This finding is consistent with the many previous studies reporting an association between ASD symptom severity and parental stress or psychological distress [9, 30-34, 36, 37, 134], and it extends previous observations on the subjective aspects of burden to the objective burden.

However, not all previous studies are in agreement. A number of studies found no relation between severity of autistic symptoms and parental stress [43, 45-47] or parental mental health problems [48]. A further study did find an association between ASD severity and parental stress, which, however, was not confirmed by multivariate analysis [49].

Several issues may account for the discrepancies between these studies regarding the association between parental burden and severity of autistic symptoms. First, given that children with ASD as a group are highly heterogeneous, differences between studies in children’s age range, ASD symptom severity, and diagnostic category significantly complicate the comparison of results across studies. Another factor potentially affecting the results lies in differences across studies in the methodology (observer-rated or self-report instruments) and the specific instruments used to measure the relevant variables. Also, studies have often analysed the contribution of only one or two child factors to the parental burden. Furthermore, differences in the samples of parents (mothers only or both maternal and paternal caregivers) make comparisons between studies difficult, as mothers and fathers may differ in terms of what child characteristics contribute most to their burden. These considerations may also account for the previously mentioned discrepancies between studies concerning the association between parental burden and other child factors such as age, adaptive functioning, and cognitive impairment.

Overall, our findings corroborate the view that the challenging symptoms of ASD contribute substantially to the experience of stress in parents. Greater symptom severity is likely to result in higher levels of dependency on parents, increasing the strain associated with caring for the child. Indeed, some studies reported an association between parental stress and two of the key diagnostic traits of ASD, namely impairments in social communication [9], and restricted or repetitive behaviours [135]. The additional contribution of the child’s functional and cognitive impairment to parental burden in some models suggests that the combination of emotional, functional, communication, and cognitive difficulties common in children with ASD affects parents more than autistic symptoms alone.

#### 4.4.2 Parent characteristics and burden

##### 4.4.2.1 Social Support

The strongest and most consistent predictor of burden was perceived social support, which was negatively associated with subjective burden, and even more strongly with the objective burden, in both mothers and fathers of children with ASD. Indeed, all previous studies except one [18] have suggested a protective role of social support against the stress associated with raising a child with ASD. Earlier studies on parents of children with autism reported that the degree of social support was negatively correlated with stress [35] and that the most powerful predictor of depression and anxiety was lower levels of social support [55]. Subsequent studies on mothers of children with ASD reported that partner and friend support were associated with maternal well-being [54], that perceived social support was negatively associated with the perceived negative impact of having a child with ASD [31], and that social support was associated with lower stress [60]. Similarly, in mothers of adolescents and adults with ASD, the quantity and valence of social support were associated with wellbeing above and beyond the impact of child behaviour problems [57]. Likewise, in a longitudinal study on mothers of young children with ASD, social support was found to be negatively associated with parenting stress [58]. As well, a study on both mothers and fathers of children with ASD reported a negative relationship between social support and stress and mental health concerns [32].

Our finding of a strong association between perceived social support and parental burden corroborates the notion that substantial support from external sources is critical to meet the huge demands associated with raising a child with a disability [5]. The key importance of social support for families facing illness and disability is attested by the finding that the only process that was consistently identified as statistically equivalent across all three study groups was the negative association between perceived social support and both objective and subjective burden in mothers. Social support from outside the nuclear family appears to be the crucial issue for parents of children with ASD, as they were found to perceive less support from the extended family and friends as compared with the other groups.

##### 4.4.2.2 Coping Strategies

The differential effectiveness of coping strategies is not a simple issue. Likely, no coping strategy is effective in all situations, and coping effectiveness is heavily dependent on the type of stressful situation that the individual faces [51]. A classical, though simplistic, distinction is made between controllable and uncontrollable stressors, expected to be better managed with problem-focused or emotion-focused coping, respectively [90].

Overall, emotion-focused coping strategies were positively associated with burden in this study. Both in mothers and fathers, disengagement coping showed a positive association with the objective and subjective burden. Also, though in mothers only, distraction coping showed a positive association with the subjective burden. These findings are consistent with previous literature, as in several studies parental burden correlated with the use of disengagement and distraction coping strategies, such as self-distraction, denial, behavioural disengagement, venting of emotions, and self-blame [18, 28, 47, 52, 53, 58].

However, in both mothers and fathers, we also found a positive association between objective burden and a problem-focused coping dimension, such as engagement coping. This finding suggests that there is no easy recipe for coping with ASD in a child based on a simple dichotomization of strategies. Parents of children with ASD need to handle both situational demands and their emotional reactions to those demands. Therefore, they have to use both problem-focused and emotion-focused coping strategies, and even the best of efforts may not be enough to solve all problems, given the severe difficulties they face.

Our finding that most coping strategies were associated with burden in some models is likely to be ascribed, at least in part, to the presence of a severely stressful situation lasting for years before the assessment, and it should be considered in light of the frequent finding across studies that coping strategies seem to have damaging rather than beneficial effects on well-being [136]. In this regard, it has been suggested that for parents confronted with a chronic condition such as ASD in a child, being able to accept the challenges that one is unable to change and to positively reframe potentially stressful events may be the most effective coping strategies, whereas not only emotion-focused coping but also problem-focused coping might not lead to satisfactory parent adjustment [48]. Consistently with this suggestion, in our study, the cognitive reframing dimension, which includes the acceptance coping strategy, was the only coping dimension that was not associated with increased burden in any model. While it was not associated with decreased burden, this may have been due to the fact that in several models it showed positive relationships with personal coping resources such as spiritual wellbeing and hardiness that were robust negative predictors of burden. Interestingly, the only instance in which hardiness was positively, rather than negatively, associated with burden was its positive indirect effect on maternal objective and subjective burden through engagement and distraction coping, respectively. This further reinforces the suggestion that, when facing chronic and difficult to manage problems such as autistic symptoms in a child, focusing forcefully and exclusively on trying to change the situation may be counter-productive [48].

##### 4.4.2.3 Personal Coping Resources

Among personal coping resources, spiritual well-being was the most consistently associated with lower burden in parents of children with ASD, with a direct negative association with the maternal subjective burden and a variety of indirect effects. Both in mothers and fathers of children with ASD, it had an indirect negative effect not only on subjective but also on objective burden through perceived social support and through disengagement coping. In mothers only, it showed a negative indirect effect on subjective burden through distraction coping. In fathers only, it had a negative indirect effect on objective burden through engagement coping. These findings suggest that parents high in spiritual wellbeing perceive their social support to be greater, and likely elicit more support from others, which in turn leads to a reduced burden. The importance of this process is corroborated by the observation that a statistically equivalent association between spiritual well-being and perceived social support was also observed in fathers of children with DS. Parents high in spiritual wellbeing seem to engage in less strenuous coping efforts, which also leads to a lower burden. Interestingly, in all models, spiritual well-being was positively associated with cognitive reframing coping, which is the only coping dimension that was not associated with increased burden, and this association was identified as statistically equivalent across the fathers of children with ASD and DS.

This pattern of results suggests that spirituality, religion, and personal principles exert a positive influence on parents’ sense of meaning and purpose in life, which is consistent with theoretical perspectives emphasizing that meaning-making is a central aspect of coping with adversity [137]. Indeed, a search for meaning has been found to help families cope with the stress associated with raising a child with ASD. Meaning-making was found to be a key topic among parents of children with autism who belonged to a support group [138]. Parents of children with high functioning ASD mentioned several benefits of their parenting experience, such as changes in their life priorities, growth in faith, spirituality, patience, and self-control [139]. In another study, some parents of children with ASD reported that they learned several important lessons, such as a shift in the meaning of life, making positive meaning of disability, becoming closer as a family, coming to appreciate small things, and experiencing a spiritual awakening or strengthening [140]. A further study reported that many parents made clear mention of the positive meaning they had found, which gave them hope to find their way and make sense out of what life has given them [141]. Therefore, our findings, together with those of qualitative studies, highlight the importance of parents of children with ASD maintaining a positive perspective and being able to make sense and find meaning in their lives.

The other personal resource investigated was hardiness, which showed mixed effects. On one hand, in mothers of children with ASD hardiness displayed a positive indirect effect on objective burden through engagement coping, and on subjective burden through distraction coping. The first finding reflects a consistent process, as the association between hardiness and engagement coping in mothers was statistically equivalent across the families of children with ASD and DS. It is consistent with the notion that individuals high in hardiness, who have a greater sense of control and mastery over life, are more likely to use problem-focused coping responses. The second finding is less straightforward to interpret. However, it should be noted that the association between hardiness and distraction coping in mothers was statistically equivalent across the families of children with ASD and DS, which suggests that it is not a negligible process. Possibly, the high perceived control of individuals high in hardiness gives them enough confidence to distract themselves from the stressful situation and attend to other duties or challenges.

On the other hand, the most consistent effect of hardiness was in the expected direction of a decrease in burden, as in both mothers and fathers of children with ASD hardiness had an indirect negative effect on both objective and subjective burden through perceived social support. This effect is similar to what was observed for spiritual well-being and suggests that parents high in hardiness tend to perceive greater social support, and likely to attract more support from others, which in turn leads to a lower burden. This finding is consistent with, and elaborates upon, a number of earlier studies. In mothers of children with autism, hardiness was found to be associated with less psychological distress [48, 142] and with lower stress [60], and it was found to be a significant mediator of the relationship between the build-up of stressors and distress [48]. Also, a study on mothers and fathers of children with ASD reported that SOC, a construct that is similar in many respects to hardiness, was associated with better mental health [56].

## 5 LIMITATIONS

This study has some limitations that should be kept in mind when looking at the findings. First, the samples were non-random, which may limit the generalizability of the results and might have introduced some sort of bias, as the levels of burden experienced by the parents may have affected their interest in and their capacity for participation in the study. Second, parent variables, such as burden, depressive and anxiety symptoms, hardiness, social support, spiritual well-being, and coping strategies, were all measured with self-report instruments. Although this choice is appropriate for most of these variables because self-reports are crucial when assessing internal states and private events, it may nevertheless raise concerns about common method bias. However, there is evidence of construct validity for the instruments used, which rules out substantial method effects. Also, there was limited conceptual overlap in the items used to measure most of the different constructs. Moreover, the inflation of relationships between variables by shared method variance is often completely offset by the attenuating effects of measurement error [143]. Third, firm conclusions about the direction of effects between variables are precluded by the cross-sectional design of the study. Fourth, given that coping is a continually changing response to continuously evolving situational demands, our reliance on a single assessment of parental coping strategies precluded inferences about the influence on the parental burden of their timing, order, combination, or duration. Finally, some potentially relevant variables, such as the birth order of the child with ASD, the quality of the couple relationship, the parental broader autism phenotype (BAP), and the severity of child problem behaviour were not explored in this study. Unfortunately, practical reasons prevented us to include the assessment of these variables into an already crowded assessment schedule, and thus we cannot comment on their relative importance among parent- and child-related factors in contributing to the parental burden. As far as parental BAP is concerned, its relevance is suggested by findings of a positive correlation of BAP with depression and parenting stress, mediated by social support and coping [134]. However, this limitation is mitigated by the fact that the study’s inclusion criteria led to the exclusion of multiplex families, which suggests that the prevalence of BAP in parents was likely low [144].

## CONCLUSION

Despite its limitations, this study confirmed that mothers and fathers of children with ASD carry a huge caregiving burden in the form of objective difficulties, subjective distress, and symptoms of depression and anxiety. This study added to our understanding of the factors that are associated with burden. Given that caregiving burden and stress may affect service utilization and reduce the effectiveness of interventions for young children with ASD [145], identifying and understanding factors related to the parental burden is important for designing effective interventions aimed at its reduction.

Health professionals working with these families should be alert to the possible presence of clinically significant depressive and anxiety symptoms in parents, which need to be promptly recognized and treated. In addition to common treatment options such as individual psychotherapy or medication, a recent study suggests that group treatments aimed at decreasing stress, depression, and anxiety, such as Mindfulness-Based Stress Reduction and positive psychology practice, may also be of help to these parents [146].

The parents who are raising children with ASD may also benefit from interventions designed to regain or improve their coping abilities through cognitive-behavioural approaches [147], from interventions such as acceptance and commitment therapy (ACT) aimed at promoting parent acceptance of negative emotions, distancing from difficult thoughts, identifying and pursuing personal values and goals [148], and also from interventions targeting parents’ abilities to manage their child’s emotional, social, and behavioural impairments and increasing parent confidence in their ability to assist their children. Indeed, the latter are the issues addressed by early intensive behavioural intervention, which has strong empirical support [149] and has been used for a long time in many places.

Finally, and probably most importantly, this study suggests that increasing the level of social support available to parents might be quite beneficial to them, as perceived social support was the strongest and most consistent negative predictor of objective and subjective burden. While the parents of children with ASD who participated in this study showed substantial resilience and ability to cope with stress, social support was found to be a really critical issue for them, as they reported lower levels of social support from family and friends than other groups. Traditional support interventions, such as respite care, may contribute to decrease parenting stress [150]. However, our findings suggest that there is a need for innovative methods for increasing social support that go beyond the commonly available peer-led or professional-led support groups. Some research suggests that support from significant others, such as friends or family members, may be more useful than support from peers in organized support groups. Also, some studies indicate that the presence of behaviour intended to be supportive that does not meet the needs of the recipient or is perceived as harmful, critical, or hostile can be counterproductive [151]. Therefore, it is plausible that interventions should aim at improving social interactions within the natural social network, and possibly at drawing significant others into therapy in an effort to improve family interactions and, thus, support. It would be important to develop methods for increasing extended family awareness and engagement, and for helping parents to connect better with their community. Studies on caregivers of persons with dementia suggest that counselling and support interventions designed to mobilise the support of naturally existing family networks may reduce caregivers’ distress and have beneficial effects on support satisfaction and objective social network variables [152]. Finally, the Internet, by providing access to online support groups and chat rooms, may provide opportunities to increase access to social support regardless of time, distance, or mobility constraints, and offer new cost-effective possibilities [153].

Future work in this area should involve prospective longitudinal studies, a few of which are already underway [27, 28, 154], to help clarify causal relationships between parental burden and child and parent factors, and the further development and evaluation of intervention approaches to reduce caregiving burden in parents of children with ASD. Such research has potential to reduce suffering in parents and the family as a whole, and possibly to improve the clinical outcome of children with ASD living in the family.

## Figures and Tables

**Fig. (1):**
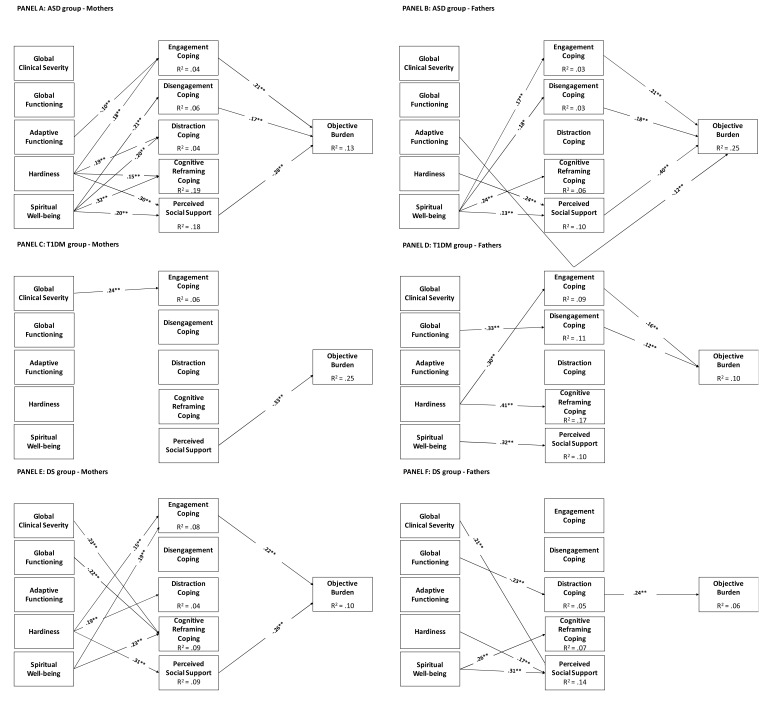
Parameter estimates for the mediational model for objective burden. Note. ** p < .01; * p < .05. Correlations between variables, and effects of covariates on major variables were estimated but not depicted for clarity.

**Fig. (2):**
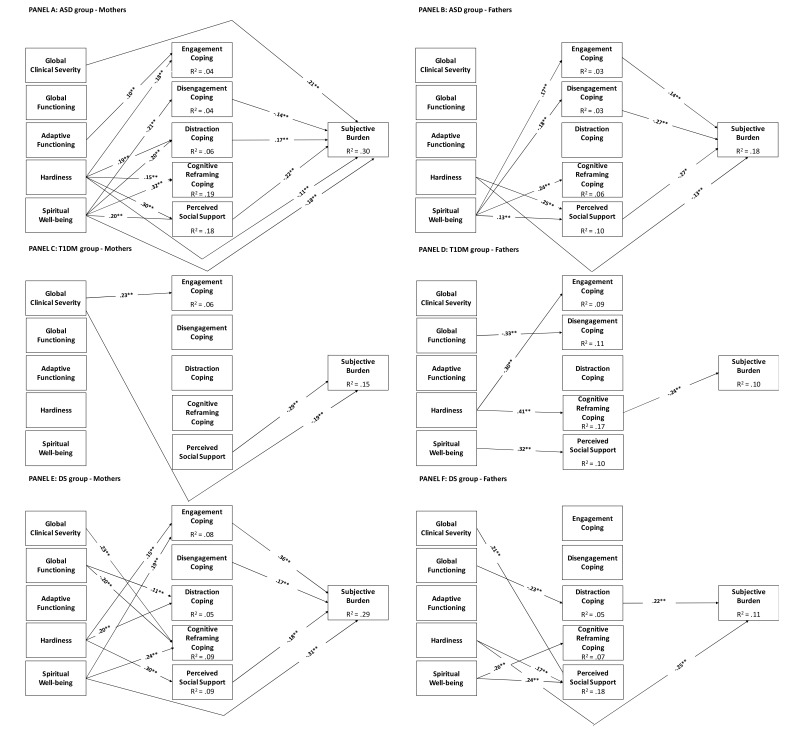
Parameter estimates for the mediational model for subjective burden. Note. ** p < .01; * p < .05. Correlations between variables, and effects of covariates on major variables were estimated but not depicted for clarity.

**Fig. (3):**
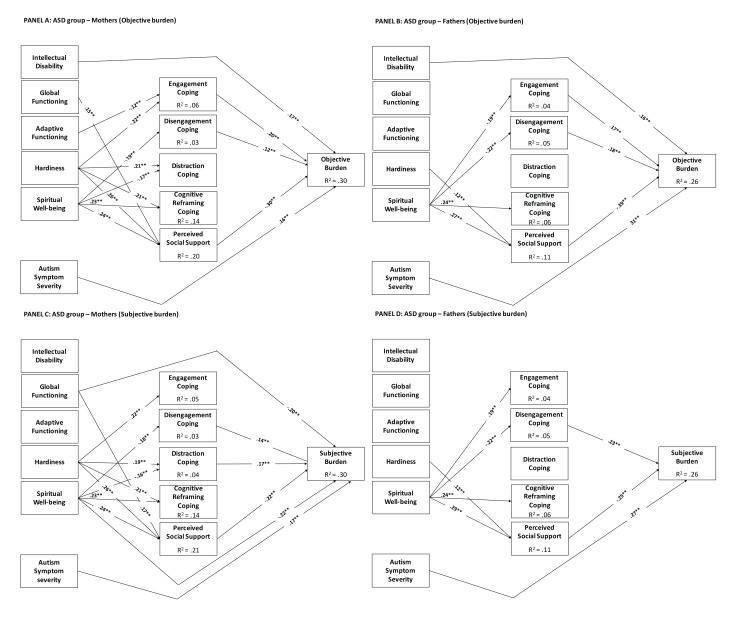
Parameter estimates for the mediational model for the parents of children with ASD only. Note. ** p < .01; * p < .05. Correlations between variables, and effects of covariates on major variables were estimated but not depicted for clarity.

**Table 1:** Sociodemographic characteristics of parents.

	**Mothers (N=644)**	**Fathers (N=536)**
**Group [N, (%)]**		
ASD	351 (54.5)	288 (53.7)
DS	140 (21.7)	115 (21.5)
T1DM	153 (23.8)	133 (24.8)
**Geographical Area [N, (%)]**		
Northern Italy	135 (21.0)	121 (22.6)
Central Italy	234 (36.3)	156 (29.1)
Southern Italy	275 (42.7)	259 (48.3)
**Patients’ Age Range [N, (%)]**		
5-8	221 (34.3)	183 (34.1)
9-12	219 (34.0)	179 (33.4)
13-17	204 (31.7)	174 (32.5)
**Age (mean ± SD)**	42.5 **±** 6.0	45.8 **±** 6.8
**Education [N, (%)]**		
Primary school	19 (3.0)	19 (3.5)
Junior high school	135 (21.0)	137 (25.6)
Senior high school	357 (55.4)	286 (53.4)
University degree	130 (20.2)	93 (17.4)
**Marital Status [N, (%)]**		
Unmarried	34 (5.3)	25 (4.7)
Married	569 (88.4)	484 (90.3)
Separated or divorced	40 (6.2)	23 (4.3)
Widowed	1 (0.2)	2 (0.4)
**Living Condition [N, (%)]**		
Lives alone	32 (5.0)	27 (5.0)
Lives with other people	608 (94.4)	506 (94.4)
**Working Status [N, (%)]**		
Employed in paid work	381 (59.2)	492 (91.8)
Housewife	217 (33.7)	3 (0.6)
Pension	5 (0.8)	11 (2.1)
Unemployed	21 (3.3)	20 (3.7)
Other	20 (3.1)	9 (1.7)

ASD = Autism Spectrum Disorder; DS = Down’s syndrome; T1DM = Type 1 diabetes mellitus.

Numbers may not sum to total due to missing data. Percentages are calculated as percentage of both available and missing data.

**Table 2:** Sociodemographic characteristics of children.

	**ASD (N=359)**	**DS (N=145)**	**T1DM (N=155)**
**Geographical Area [N, (%)]**	–	–	–
Northern Italy	82 (22.8)	9 (6.2)	47 (30.3)
Central Italy	141 (39.3)	64 (44.1)	36 (23.2)
Southern Italy	136 (37.9)	72 (49.7)	72 (46.5)
**Sex [N, (%)]**	–	–	–
Male	309 (86.1)	87 (60.0)	77 (49.7)
Female	50 (13.9)	58 (40.0)	77 (49.7)
**Age Range [N, (%)]**	–	–	–
5-8	140 (39.0)	49 (33.8)	39 (25.2)
9-12	113 (31.5)	49 (33.8)	60 (38.7)
13-17	106 (29.5)	47 (32.4)	56 (36.1)
**Age (mean ± SD)**	9.9 **±** 3.7	10.3 **±**3.9	11.0 **±**3.5
**Educational Support at School [N, (%)]**	–	–	–
Yes	329 (91.6)	138 (95.2)	0 (0.0)
No	19 (5.3)	7 (4.8)	155 (100.0)
**Public Financial Support for School Attendance [N, (%)]**	–	–	–
Yes	123 (34.3)	39 (26.9)	40 (25.8)
No	223 (62.1)	106 (73.1)	115 (74.2)
**Invalidity Benefit [N, (%)]**	–	–	–
Yes	234 (65.2)	128 (88.3)	2 (1.3)
No	107 (29.8)	11 (7.6)	147 (94.8)
**Parental Work Facilitation [N, (%)]**	–	–	–
Yes	187 (52.1)	82 (56.6)	12 (7.7)
No	159 (44.3)	58 (40.0)	143 (92.3)
**Parental Membership in Family Associations [N, (%)]**	–	–	–
Yes	121 (33.7)	103 (71.0)	74 (47.7)
No	226 (63.0)	41 (28.3)	81 (52.3)
**Single-parent Family [N, (%)]**	–	–	–
Mother only	10 (2.8)	2 (1.4)	2 (1.3)
Father only	6 (1.7)	1 (0.7)	0 (0.0)
**Number of Other Siblings [N, (%)]**	–	–	–
0	97 (27.0)	30 (20.7)	37 (23.9)
1	205 (57.1)	71 (49.0)	93 (60.0)
2	34 (9.5)	28 (19.3)	21 (13.5)
3	10 (2.8)	9 (6.2)	2 (1.3)
4	2 (0.6)	6 (4.1)	1 (0.6)
**Parental Full-time Employment [N, (%)]**	–	–	–
Both parents	97 (27.0)	38 (26.2)	49 (31.6)
Father only	207 (57.7)	91 (62.8)	87 (56.1)
Mother only	6 (1.7)	1 (0.7)	8 (5.2)

ASD = Autism Spectrum Disorder; DS = Down’s syndrome; T1DM = Type 1 diabetes mellitus.

Numbers may not sum to total due to missing data. Percentages are calculated as percentage of both available and missing data.

**Table 3:** Clinical characteristics of children.

	**ASD (N=359)**	**DS (N=145)**	**T1DM (N=155)**	Significant Between-group Differences *
**Duration of Illness From First Diagnosis** (mean ± SD)	6.1 **±** 3.6	10.3 **±**3.9	6.2 **±** 3.2	DS > ASD and T1DM (p<0.001)
**Current Treatment [N, (%)]**	–	–	–	–
Pharmacotherapy	85 (23.7)	27 (18.6)	155 (100.0)	not tested
Psychomotor Interventions	158 (44.0)	66 (45.5)	0 (0.0)	not tested
Speech therapy	184 (51.3)	94 (64.8)	0 (0.0)	not tested
Psychoeducation	132 (36.8)	31 (21.4)	1 (0.6)	not tested
Occupational therapy	22 (6.1)	5 (3.4)	0 (0.0)	not tested
Cognitive behavioural therapy	23 (6.4)	2 (1.4)	2 (1.3)	not tested
Family therapy	18 (5.0)	1 (0.7)	1 (0.6)	not tested
Alternative and complementary therapies	75 (20.9)	7 (4.8)	0 (0.0)	not tested
**CGI-S** (mean ± SD)	4.2 **±** 1.2	3.8 **±** 0.8	3.2 **±** 1.6	ASD > DS > T1DM (all p<0.001)
**Intellectual disability [N, (%)]**	–	–	–	–
Absent	92 (25.6)	1 (0.7)	155 (100.0)	T1DM > ASD > DS (all p<0.001)
Mild	94 (26.2)	52 (35.9)	0 (0.0)	ASD and DS > T1DM (p<0.001)
Moderate	107 (29.8)	75 (51.7)	0 (0.0)	DS > ASD > T1DM (all p<0.001)
Severe	50 (13.9)	14 (9.7)	0 (0.0)	ASD and DS > T1DM (p<0.001)
Profound	15 (4.2)	3 (2.1)	0 (0.0)	ASD > T1DM (p<0.05)
**CARS** (mean ± SD)	36.5 ± 6.6	22.8 ± 4.2		ASD > DS (p<0.001)
**ADOS Communication** (mean ± SD)	5.8 **±** 2.0	-	-	
**ADOS Social** (mean ± SD)	9.7 **±** 2.8	-	-	
**CGAS** (mean ± SD)	45.5 **±** 15.3	55.7 **±** 13.1	89.9 **±** 5.0	ASD > DS > T1DM (all p<0.001)
**VABS age-equivalent scores** (months)	–	–	–	–
Communication (mean ± SD)	51.2 **±** 37.5	55.8 **±** 29.9	132.3 **±** 32.7	ASD < DS < T1DM (all p<0.001)
Daily living skills (mean ± SD)	49.6 **±** 29.5	54.3 **±** 25.5	144.44 **±** 7.0	ASD < DS < T1DM (all p<0.001)
Socialization (mean ± SD)	34.2 **±** 22.6	51.1 **±** 24.7	149.0 **±** 51.2	ASD < DS < T1DM (all p<0.001)
Motor skills (mean ± SD)	47.2 **±** 14.0	42.6 **±** 13.2	62.9 **±** 5.4	DS < ASD (p<0.05) < T1DM (p<0.001)

ASD = Autism Spectrum Disorder; DS = Down’s syndrome; T1DM = Type 1 diabetes mellitus; CGI-S = Clinical Global Impression-Severity; ADOS = Autism Diagnostic Observation Schedule; CGAS = Children’s Global Assessment Scale; VABS = Vineland Adaptive Behavioural Scale. Numbers may not sum to total due to missing data. Percentages are calculated as percentage of both available and missing data. * after adjustment for age and sex

**Table 4:** Parental burden and presence of significant depressive and anxiety symptoms by diagnostic group.

–	**ASD**		**DS**		**T1DM**		Significant Between-group Differences *
–	**Mothers**	**Fathers**	**Mothers**	**Fathers**	**Mothers**	**Fathers**	–
**FPQ** (mean item score ± SD)	–	–	–	–	–	–	–
Objective burden	1.9±0.7	1.8±0.7	1.6±0.6	1.5±0.5	1.5±0.5	1.4±0.3	ASD > DS > T1DM (all p<0.001) for both mothers and fathers
Subjective burden	2.0±0.6	1.8±0.6	1.6±0.5	1.5±0.4	1.6±0.4	1.5±0.4	ASD > DS > T1DM (all p<0.001) for both mothers and fathers
Adverse impact on other children under age 12 years	3.2±1.5	3.1±1.5	2.6±1.2	2.5±1.0	2.3±0.9	2.2±0.8	ASD > DS and T1DM (all p<0.01) for both mothers and fathers
Adverse impact on parental work	2.8±1.0	2.7±1.1	2.5±0.7	2.4±0.9	2.3±0.6	2.1±0.5	Mothers: ASD > DS (p<0.05) and T1DM (p<0.01) Fathers: ASD > DS (p<0.05) > T1DM (p<0.05)
Child health-related expenses (thousand euros, last year)	1.36±4.05	1.75±5.72	0.72±1.81	0.62±1.71	0.08±0.37	0.06±0.25	Mothers: ASD > T1DM (p<0.001) Fathers: ASD > DS (p<0.05) and T1DM (p<0.01)
**Time devoted to the child** (hours per week) (mean ± SD)	43.0±17.7	26.2±12.3	41.8±16.2	25.6±14.2	36.3±14.6	23.8±11.3	Mothers: T1DM < DS (p<0.01) and ASD (p<0.001)
**Significant depressive and anxiety symptoms on the GHQ-12** (N, *%*)	113 *(33.0)*	85 *(29.9)*	29 *(21.0)*	16 *(14.3)*	40 *(26.7)*	31 *(23.8)*	Fathers: ASD > DS (p<0.01)

ASD = Autism Spectrum Disorder; DS = Down’s syndrome; T1DM = Type 1 diabetes mellitus; FPQ = Family Problems Questionnaire; GHQ = General Health Questionnaire; * after adjustment for age and for child’s age and sex

**Table 5:** Parental coping, social support, hardiness, and spiritual wellbeing by diagnostic group.

–	**ASD**		**DS**		**T1DM**		Significant Between-group Differences *
–	**Mothers**	**Fathers**	**Mothers**	**Fathers**	**Mothers**	**Fathers**	–
**Brief COPE** (mean ± SD)	–	–	–	–	–	–	–
Engagement	23.3±4.8	21.7±4.3	22.6±5.1	20.8±4.3	20.8±5.2	19.6±5.3	Mothers: T1DM < ASD (p<0.001) and DS (p<0.01) Fathers: T1DM <ASD (p<0.01)
Disengagement	7.7±2.4	7.8±2.3	7.5±2.0	7.5±2.1	8.1±2.4	8.2±2.4	Fathers: T1DM > DS (p<0.05)
Distraction	17.3±4.0	15.6±3.5	16.4±4.1	15.3±4.2	15.9±4.0	15.0±4.2	Mothers: T1DM < ASD and DS (all p<0.01)
Cognitive Reframing	17.2±3.8	16.0±3.4	17.5±3.7	15.9±3.8	16.1±4.2	15.0±4.1	Mothers: ASD >T1DM (p<0.01) Fathers: ASD >T1DM (p<0.05)
**MSPSS** (mean ± SD)	–	–	–	–	–	–	–
Total score	61.6±14.7	63.4±12.2	67.3±11.9	65.1±11.2	67.4±12.6	68.7±10.3	Mothers: ASD <DS and T1DM (all p<0.001) Fathers: T1DM > ASD (p<0.001) and DS (p<0.05)
Significant Other	17.0±4.1	17.8±3.5	18.2±3.2	18.3±3.0	17.8±3.5	18.2±2.8	Mothers: ASD <DS (p<0.01)
Family	15.9±4.4	16.7±3.6	17.3±3.7	16.9±3.5	17.5±3.7	18.3±2.5	Mothers: ASD < T1DM and DS (all p<0.01) Fathers: T1DM > ASD (p<0.001) and DS (p<0.01)
Friends	13.4±4.8	13.0±4.2	14.9±4.4	13.7±4.3	15.4±3.9	15.0±4.1	Mothers: ASD < T1DM (p<0.001) and DS (p<0.01) Fathers: T1DM > ASD (p<0.001) and DS (p<0.05)
**DRS-15** (mean ± SD)	27.7±5.5	27.9±5.2	27.8±5.5	27.8±5.7	27.4±5.0	27.7±5.2	–
**WHOQOL-100 SRPB** (mean ± SD)	69.2±21.4	70.9±18.8	75.3±18.4	71.1±17.7	71.7±17.7	73.4±18.3	–

ASD = Autism Spectrum Disorder; DS = Down’s syndrome; T1DM = Type 1 diabetes mellitus; FPQ = Family Problems Questionnaire; GHQ = General Health Questionnaire; DRS = Dispositional Resilience Scale; MSPSS = Multidimensional Scale of Perceived Social Support; WHOQOL-100 SRPB = 100-item version of the World Health Organization Quality Of Life questionnaire, Spirituality Religion and Personal Beliefs facet subscale * after adjustment for age and for child’s age and sex
